# Fokaler Haarverlust bei einem blondhaarigen Jungen – Diagnose mittels Trichoskopie

**DOI:** 10.1007/s00105-020-04656-6

**Published:** 2020-07-28

**Authors:** Christine Prodinger, Verena Ahlgrimm-Siess

**Affiliations:** Universitätsklinik für Dermatologie und Venerologie, Paracelsus Medizinische Privatuniversität, Müllner Hauptstr. 48, 5020 Salzburg, Österreich

In der Diagnostik von Haarerkrankungen hat sich die dermatoskopische Untersuchung von Haaren und Kopfhaut (Trichoskopie) als hilfreiches nichtinvasives Tool etabliert. Wie der folgende Fall zeigt, gibt es allerdings Besonderheiten zu beachten wie z. B. bei der Untersuchung von blonden und nicht pigmentierten Haaren.

## Anamnese

Ein 12-jähriger, blondhaariger Junge wird in unserer Ambulanz vorstellig, da seit etwa 7 Monaten langsam größenprogrediente Areale mit Haarlichtung am Kapillitium bestehen. Der Patient gibt einen leichten Juckreiz im Bereich der betroffenen Stellen an, sonst sind anamnestisch keine weiteren Beschwerden, Allergien oder Vorerkrankungen erhebbar, und der Patient kommt in seiner näheren Umgebung nicht mit (Haus‑)Tieren in Kontakt.

## Klinischer Befund

**Figure 1:**
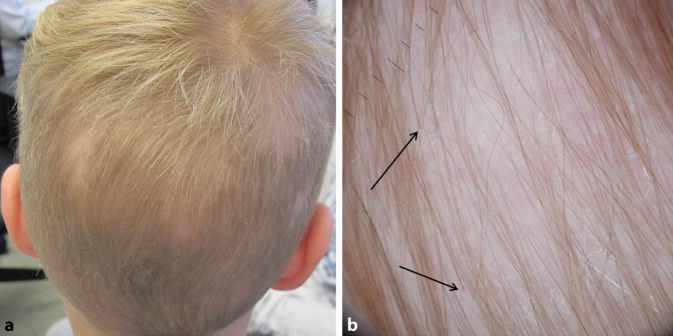

Klinisch zeigen sich mehrere, schlecht abgrenzbare Areale mit reduzierter Haardichte und wenigen kurzen Haaren (Abb. 1a). Der Zupftest ist negativ.

## Trichoskopischer Befund

Bei der trichoskopischen Untersuchung mit Immersionsmedium zeigen sich neben einer Reduktion der Haardichte mehrere kurze, pigmentierte Haare mit stumpfen Enden (Abb. 1b), einem relativ unspezifischen Befund entsprechend.

**Figure 2:**
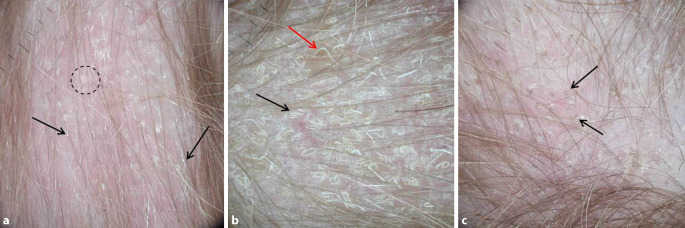

Ohne Verwendung eines Immersionsmediums („dry dermoscopy“) können weitere Haarschaftveränderungen dargestellt werden, wie z. B. stark reflektierende, weißliche Querbänder im Verlauf und an den Enden kurzer Haarschäfte (Abb. 2a) sowie nicht pigmentierte Haare mit zum Teil bizarren Formveränderungen (Abb. 2b).

## Weiteres Vorgehen

Zur weiteren Abklärung wird eine lichtmikroskopische Untersuchung epilierter Haare durchgeführt.

## Wie lautet Ihre Diagnose?

## Weiterführende Untersuchungen

Nach Zugabe von Kalilauge können lichtmikroskopisch Pilzelemente in den Haarschäften dargestellt werden. Dieser Befund wird durch die positive mykologische Kultur und den molekulargenetischen Nachweis von *Trichophyton tonsurans* bestätigt.

## Diskussion

Die Verdachtsdiagnose einer Tinea capitis wird neben dem lichtmikroskopischen Nachweis befallener Haare durch den Erregernachweis mittels mykologischer Kultur oder molekularbiologischer Methoden bestätigt, die in der Regel eine Klassifizierung des Erregers innerhalb weniger Tage bis Wochen ermöglichen. Da die diagnostische Empfindlichkeit des mikroskopischen Befundes gering ist, der kulturelle Nachweis zeitverzögert erfolgt und molekularbiologische Methoden nicht ubiquitär verfügbar sind, kommt der klinischen Diagnostik eine besondere Bedeutung zu.

Neben dem gemeinsamen Vorliegen von Alopezie, Schuppung und Inflammation bei der klinischen Untersuchung kann eine Fluoreszenz im Wood-Licht (z. B. hellgrüne Fluoreszenz bei einigen *Microsporum*-Arten) auf eine infektiöse Genese des Haarausfalls hinweisen. Typische Haarschaftveränderungen in der Trichoskopie der Tinea capitis (Tab. 1) ermöglichen die Abgrenzung zu anderen Haarerkrankungen und werden in der Literatur auch mit dem Vorliegen bestimmter Erreger in Verbindung gebracht [1–4]. Das Verschwinden der Haarveränderungen unter Therapie kann den Therapieerfolg bei Verlaufskontrollen anzeigen [5, 6].

**Table 1:** 

Zeichen	Beschreibung	Tinea capitis	Alopecia areata	Trichotillomanie
Kommahaare	Gebogene Haare mit stumpfen Enden, ähnlich dem „Komma“	+	−	−
Korkenzieherhaare	Mehrfach gewundene Haare	+	−	−
Morse-Code-Haare	Reflektierende transversale Streifen im Haarschaft (= Bruchstellen)	+	−	−
Blockhaare	Kurze Haare mit scharf abgegrenztem distalem Ende	++	+/−	+/−
I‑Haare	Blockhaare mit akzentuiertem dunklem Haarende	+	−	+/−
Zickzackhaare	An mehreren Stellen abgewinkelte Haarschäfte	+	+/−	−
„Black dots“	Schwarze Punkte	+	++	+

Morphologisch unterscheidet man 3 Arten einer möglichen Haarinfektion:

*Ektothrix* – Pilzhyphen und Sporen bedecken die Außenseite der Haare bis zur Keratinisierungszone, bei Progression kann eine transversale Perforation auftreten (z. B. bei Infektion durch *Microsporum canis*). Trichoskopisch führt die Sporenakkumulation am Haarschaft zunächst zu Aufhellungen, in späteren Stadien zu Querbändern und Bruchstellen mit dem Vorliegen sog. „Zickzack-“ und „Morse-Code-Haare“.

*Endothrix* – Pilzhyphen und Sporen dringen in das Haar ein und verursachen eine Schädigung des Schafts, die sich trichoskopisch als Haardeformierung manifestiert (z. B. als „Komma-“ oder „Korkenzieherhaar“).

*Kombination Ektothrix/Endothrix* – Ektotricher und endotricher Befall treten nebeneinander auf mit entsprechenden trichoskopischen Zeichen aus beiden Gruppen (z. B. bei Infektion durch *Trichophyton tonsurans*; Abb. 2).

Immersionsmedien (z. B. Alkohol, Wasser) sind in der Dermatoskopie zwar nützlich, um die Reflexion von Licht zu reduzieren und eine verbesserte Darstellung von Strukturen unterhalb der Hautoberfläche zu erreichen, bei der Untersuchung von Haaren und Kopfhaut ist aber zunächst der sog. „dry dermoscopy“ mit polarisiertem Licht der Vorzug zu geben; hellblonde oder nicht pigmentierte Haare werden ebenso wie Schuppen bei Verwendung eines Immersionsmediums durchsichtig und können nicht mehr beurteilt werden. Ist eine genauere Darstellung von Strukturen der Kopfhaut (z. B. Gefäßmorphologie) erforderlich, kann im Anschluss an die Untersuchung der Haare ein Immersionsmedium verwendet werden.

**Diagnose:** Tinea capitis

Dieses Phänomen zeigt sich auch bei unserem Patienten: in der „dry dermoscopy“ eines alopezischen Herdes finden sich zahlreiche „Morse-Code-Haare“ (Abb. 2a). Bei Verwendung eines Immersionsmediums kommen an derselben Lokalisation nur einzelne kurze pigmentierte Haare mit stumpfen Enden zur Darstellung (Abb. 1b). Diese können neben der Tinea capitis auch bei der Alopecia areata und der Trichotillomanie, also bei allen im Kindesalter häufig auftretenden fokalen Haarausfallserkrankungen, gefunden werden. In der „dry-dermoscopy“ anderer Herde zeigen sich weitere, für Pilzinfektionen typische Haarschaftveränderungen, nämlich „Kommahaare“ (Abb. 2c) sowie weiße „Korkenzieher‑“ und „Zickzackhaare“ (Abb. 2b), die bei Verwendung eines Immersionsmediums nicht dargestellt werden können. Diese Haarschaftveränderungen ermöglichen die Abgrenzung von anderen im Kindesalter auftretenden fokalen Alopezien.

Das „Verschwinden“ hellblonder und nicht pigmentierter Haarschäfte bei Verwenden eines Immersionsmediums könnte erklären, warum in der Literatur bislang nur dermatoskopische Fallberichte dunkelhaariger Patienten mit Tinea capitis veröffentlicht wurden. Der von uns vorgestellte Fall zeigt, dass trichoskopische Zeichen einer Tinea capitis bei richtiger Anwendung auch bei hellhaarigen Patienten gut darstellt werden können.

## Fazit für die Praxis

- Die Trichoskopie erlaubt eine rasche, nichtinvasive Diagnose der Tinea capitis.
- In der Trichoskopie finden sich neben einer Reduktion der Haardichte typische Haarschaftveränderungen („Kommahaare“, „Korkenzieherhaare“, „Morse-Code-Haare“ und „Zickzackhaare“) sowie stumpfe Enden nach Haarbruch.
- Die „dry dermoscopy“ ohne Immersionsmedium kann entscheidend zur Erkennung typischer trichoskopischer Merkmale bei hellblonden und nicht pigmentierten Haaren beitragen und sollte routinemäßig durchgeführt werden.
